# Building an Egocentric-to-Allocentric Travelling Direction Transformation Model for Enhanced Navigation in Intelligent Agents

**DOI:** 10.3390/s25113540

**Published:** 2025-06-04

**Authors:** Zugang Chen, Haodong Wang

**Affiliations:** 1 Aerospace Information Research Institute, Chinese Academy of Sciences, Beijing 100094, China; chenzg@aircas.ac.cn; 2 School of Computer and Artificial Intelligence, Zhengzhou University, Zhengzhou University Main Campus, Zhengzhou 450001, China

**Keywords:** half-adder unit, biomimetic neural network, coordinate transformation, intelligent agent, central complex

## Abstract

Many behavioral tasks in intelligent agent research involve working with mathematical vectors. While traditional methods perform well in some cases, they struggle in complex and dynamic environments. Recently, bionic neural networks have emerged as a novel solution. Studies on the Drosophila central complex have revealed that these insects use neural signals from the ellipsoid body and fan to track allocentric travel angles and update spatial awareness during movement, a process that heavily relies on directional vector manipulation. Our model accurately replicates the neural connectivity of the Drosophila central complex, drawing inspiration from the half-adder unit to efficiently encode and process spatial direction information. This framework significantly enhances the accuracy of coordinate transformations while increasing adaptability and resilience in challenging environments. Our experimental results demonstrate that the bionic neural network outperforms traditional methods, delivering superior precision and robust generalizability within the coordinate system.

## 1. Introduction

Determining one’s direction from self-motion cues is fundamental for animal navigation. For example, desert ants can use “dead reckoning” (path integration) to track their path [1,2], as can black-belly ants [3,4,5]. For accurate navigation, the angular course of the insect brain needs to be adjusted in real time on self-motion cues. Specifically, the brain needs to transform translational velocity signals into a world-centric coordinate system. By integrating its estimation of body-centric translational direction with its estimation of world-centric heading direction, the brain can predict an animal’s direction of travel in a world-centric coordinate system.

The insect central complex (CX), a conserved neural architecture across arthropods, has emerged as the neurobiological substrate for multisensory integration and coordinate transformation [6]. In *Drosophila melanogaster*, the CX’s tripartite structure—comprising the protocerebral bridge (PB), fan-shaped body (FB), and ellipsoid body (EB)—forms a polarized neural compass that integrates (1) idiothetic cues from haltere-mediated angular velocity sensors, (2) optic flow-derived translational vectors [7], and (3) polarized light patterns from the dorsal rim area [8]. Crucially, the recent connectomic mapping of Drosophila CX [6] revealed columnar projection neurons that implement a biologically plausible coordinate transformation algorithm through their topographically organized synapses, exhibiting striking parallels with artificial neural networks.

In recent years, biologically inspired neural networks and intelligent algorithms have demonstrated tremendous potential in both real-time simulation and neurological disorder research. For example, Beaubois et al. introduced a method that utilizes biomimetic spiking neural networks for real-time simulation and hybrid studies, offering a novel tool for exploring neurological diseases [9]. In addition, a review on biomimicry and intelligent algorithms highlighted the importance and diverse applications of these techniques in various practical settings [10]. Nonetheless, existing research still falls short in applying self-referenced-to-external reference coordinate transformation and navigation systems. Consequently, we aim to develop a coordinate transformation model that converts self-referenced coordinates to external reference coordinates with both high accuracy and robustness, thereby providing a more efficient solution for intelligent navigation systems. This neural computation faces a key challenge: the nonorthogonal transformation between body axes and world-centered coordinates. For example, when a Drosophila fly needs to move toward a specific environmental target during flight, it must continuously adjust its trajectory in relation to its surroundings [11,12]. Specifically, EB neurons maintain a persistent activity bump representing the heading direction, whereas the PB circuit performs vector rotation via phase-coupled oscillations.

Understanding this coordinate transformation mechanism holds dual scientific significance: it not only elucidates the neural basis of animal spatial cognition but also inspires novel paradigms for bioinspired navigation systems and neuromorphic computing architectures [13]. This study achieves precise motion direction conversion across reference frames via a computational model that biomimetically simulates the neural circuitry of the Drosophila central complex. Our proposed ellipsoid body–protocerebral bridge (EB-PB) encoding–decoding algorithm successfully encodes egocentric motion vectors into bionic neural networks while enabling accurate allocentric direction decoding.

The paper’s primary contributions are as follows:

(1) Developing a half-order-like directional computation model: This model establishes a theoretical framework for coordinate transformation through the synergistic processing of directional vectors.
(2) A novel head-direction encoding mechanism is proposed: This mechanism encodes spatial direction vectors into discrete signals compatible with neural networks, providing a mathematical foundation for complex spatial navigation and positional awareness.
(3) Designing a Drosophila-inspired network architecture: Based on the biomimetic modeling of Drosophila central complex circuits, this network incorporates an EB-PB structure, achieving biologically egocentric-to-allocentric (ego–allo) coordinate transformation.

The model demonstrates remarkable robustness and computational precision in coordinate transformation, aiming to provide a more efficient, accurate, and biologically plausible neural signal processing solution.

The remainder of this paper is structured as follows. In Section 2, we delve into the state of the art. Section 3 presents the overarching design concepts and methodologies. This particular section focuses on the utilization of bionic neural networks for transforming the system’s ego–allo coordinates. Section 4 evaluates the innovations and shortcomings of our research. Finally, the concluding section summarizes the research findings.

## 2. State of the Art

Modeling brain activity patterns is fundamental to understanding the computational mechanisms of the nervous system [14]. The quantitative modeling of neural signals underpins investigations into the complex functions of the brain across disciplines such as neuroscience, intelligent interaction, and bionic mechanical engineering [15,16]. Among these challenges, simulating coordinate system transformations—centered on the self (egocentric) and others (allocentric)—represents a fundamental hurdle in studying biological navigation systems.

Traditional methods for modeling brain activity typically depend on linear models [17], such as Principal Component Analysis (PCA) [18] and Canonical Correlation Analysis (CCA) [19]. These approaches extract key features from signals through dimensionality reduction, partially revealing the structural patterns of brain activity [20]. Nevertheless, they face significant limitations when processing high-dimensional, nonlinear, and dynamic brain signals. These methods often struggle to capture the complex information within the signals, leading to information loss and restricting the effectiveness of the models in practical applications.

To develop a computational model capable of solving the egocentric-to-allocentric coordinate transformation, researchers have proposed a range of methods. These methods are generally classified into three categories: those based on mathematical models, those inspired by bionic principles, and other innovative approaches.

The ongoing progression and refinement of mathematical models have served as a pivotal source of inspiration for advancements in motion-direction coordinate transformation models. Nasry introduced a methodology for coordinate transformation across various reference frames, and he implemented a novel approach for such transformations by leveraging the properties of geometric algebra, including vector reflection and rotation. On the basis of Clifford algebraic properties, he combined vectors from different planes into a mathematically coherent system that realizes comprehensive coordinate changes [21]. Akyilmaz built a computational model for coordinate transformations utilizing the total least squares (TLS) estimation method for converting point coordinates from one coordinate system to another. The TLS method allows errors in both the observations and the design matrix to be considered, providing a more realistic estimate of the transformation parameters [22]. Felus and Burtch presented a weighted total least squares (WTLS) approach for coordinate transformation. Their model extends the traditional TLS method by incorporating individual weights for observations, leading to more accurate results when dealing with heteroscedastic measurement errors. The mathematical framework they developed provides a rigorous solution for cases where both coordinate sets contain random errors [23].

Recent advances in neuroscience have further illuminated the neural circuits underlying coordinate transformations [11,24], providing valuable insights into neuroscience and computing. The discovery of specific neural circuits in insects, particularly the central complex, has revolutionized our understanding of spatial processing [25]. The relatively simple yet efficient navigation system of Drosophila has emerged as a promising model for coordinate transformation. For example, Lyu et al. conducted a comprehensive study to demonstrate the mechanisms by which the central complex of *Drosophila melanogaster* performs vector arithmetic. Specifically, they elucidated the neural circuits that map two-dimensional (2D) vectors onto sinusoidal activity patterns, enabling ego–allo transformation within the insect’s brain. This research contributes significantly to our understanding of the neural basis of spatial computation in Drosophila [26]. Similarly, Sun et al. developed a decentralized navigation model using three interconnected ring attractor networks. The first ring encodes head direction through reciprocally inhibited neurons, the second ring processes velocity inputs through direction-selective cells, and the third ring combines these signals via multiplicative integration. Their key innovation was implementing coordinate transformation through systematic phase shifts between these rings, controlled by carefully tuned synaptic weights [27]. Pisokas et al. further demonstrated that differences in neuronal inhibition patterns allow the Drosophila circuitry to respond more rapidly to changes in course. They developed a three-layer neural structure for processing course changes: the first layer consists of 16 compass-sensitive neurons that respond to polarized light patterns; the middle layer comprises 8 interneurons that receive triangulated, weighted inputs; and the final layer integrates these signals nonlinearly to produce a globally referenced directional output [28]. Hulse et al. constructed a detailed circuit model of the central complex on the basis of electron microscopy data. Their model achieves coordinate frame transformation through three key components: (1) a ring attractor network in an ellipsoid containing 32 locally excited, globally inhibited ellipsoid body–protocerebral bridge (E-PG) neurons; (2) a parallel array of 16 protocerebral–ellipsoid body–nodulus (P-EN) neurons providing speed inputs; and (3) columnar neurons that interconnect these structures with phase-locked activity. This coordinated shift is driven by a precisely timed activation pattern in which P-EN neurons systematically modulate E-PG activity in accordance with movement direction [6]. In another approach, Le Moël et al. developed a vector-based navigation model comprising three distinct neural populations: an array of 360 compass neurons for directional reference, a population of speed-sensitive neurons encoding movement velocity, and integrator neurons that combine these signals. Their computational mechanism, which combines compass and speed inputs via multiplicative interactions weighted sinusoidally, enables the network to maintain and update spatial vectors across different reference frames [29]. Burgess (2006) developed a comprehensive model of ego–allo transformation incorporating head direction cells for orientation reference, place cells for allocentric positioning, and transformation circuits linking viewpoint-dependent and viewpoint-independent representations [30]. This model explains how the brain switches between reference frames during navigation.

Beyond these approaches, Byrne et al. (2007) proposed a computational model of ego–allo transformation that features a parietal window for representing egocentric spatial information, a temporal window for maintaining allocentric representations, and a transformation circuit that utilizes head direction signals to mediate reference frame conversion [31]. Similarly, Mou et al. (2004) implemented an intrinsic reference frame model based on three main components: (1) principal axis extraction from environmental geometry, (2) orientation alignment on the basis of salient features, and (3) systematic testing of spatial memory organization [32]. Their research provided valuable insights into how spatial memories are structured and transformed between different reference frames.

Various methodological approaches are currently being explored in ego–allo coordinate transformation research, each with unique strengths and limitations. Traditional mathematical models, such as geometric algebra and TLS/WTLS, demonstrate mathematical rigor in coordinate transformations but have difficulty handling nonlinear noise interference in dynamic environments [21,22,23]. In contrast, existing biomimetic models—such as ring attractor networks [27] and simulations of the Drosophila central complex [6,26]—successfully replicate biological navigation mechanisms but are limited by the noise sensitivity of continuous phase coding and high computational costs.

The core innovation of our study lies in introducing a novel hybrid computational framework that, for the first time, combines the deterministic logic of digital half-adder circuits (which separates carry and summation functions) with the sparse coding properties of the Drosophila EB-PB circuit. This integration achieves a balanced trade-off between high precision and low resource consumption. For example, Varga and Ritzmann proposed a model in which phase coupling is achieved via synaptic weights, relying on the synchronization of continuous neural activity for coordinate transformation [33]. However, their encoding method is sensitive to noise and cannot handle dynamic alignment between nonorthogonal reference frames.

Similarly, Turner-Evans and colleagues updated headings by driving phase shifts in ring attractors through velocity inputs [34]. However, their model depends on precise velocity signal inputs, and its computational complexity increases exponentially with the number of rings.

In previous work, deep neural network methods have achieved significant results in nonlinear distortion removal and underwater acoustic communication. For example, Ma et al. [35] proposed a deep neural network-based approach for nonlinear distortion removal that effectively mitigates the peak-to-average power ratio issue, and its efficacy was demonstrated in underwater acoustic communication experiments. Similarly, Zuberi et al. [36] designed a deep neural network-based downlink nonorthogonal multiple access receiver for underwater communication, highlighting the remarkable ability of deep networks to process complex signals. Furthermore, Sinha et al. [37] conducted research on biomimetic tunable devices inspired by biological sensors, providing experimental evidence for optimizing device performance from a physiological standpoint. Despite the distinct strengths of these methods, they often face challenges such as high computational complexity or insufficient robustness in specific application scenarios. These limitations guide us toward developing a high-efficiency coordinate transformation model inspired by the central complex (CX), which promises to address these issues in intelligent navigation systems.

Building on these insights, it would be valuable to explore comparative evaluations of our proposed model against conventional deep learning techniques, particularly in domains where real-time performance and robustness are critical. This comparison might reveal additional opportunities for improving algorithmic efficiency, further broadening the scope of applications.

Our proposed bionic computing model introduces a half-adder-based coordinate transformation model, that seamlessly integrates integrating digital computation principles with biological neural circuits. The model architecture features an EB-PB encoder–decoder system, where the encoder translates self-referential motion signals into digital neural representations via phase–amplitude encoding, and the decoder transforms these signals into noncentralized directional outputs via a bionic half-adder mechanism. This innovative synergy between biological and computational principles enables highly precise coordinate transformations while preserving structural simplicity and computational efficiency. The modular design, centered around the half-adder framework, not only guarantees accurate directional conversion but also lays a robust foundation for the development of more advanced computational functionalities. By employing bionic encoding and decoding algorithms, our model achieves reliable coordinate transformations with minimal computational resource requirements, making it particularly well suited for practical applications in navigation systems.

## 3. Methods

### 3.1. General Idea

This section introduces the transformation of the bionic course coordinate system utilizing a half-adder-like structure. The ego–allo coordinate system is initially defined on a two-dimensional Euclidean plane, yielding the system’s course vector and allocentric coordinate vector, represented as sinusoidal curves. The amplitude and phase of these curves correspond to the vector’s length and angle, respectively. To encode the directional vectors represented by the sine curves, we propose a novel coding scheme.

**Figure 1:**
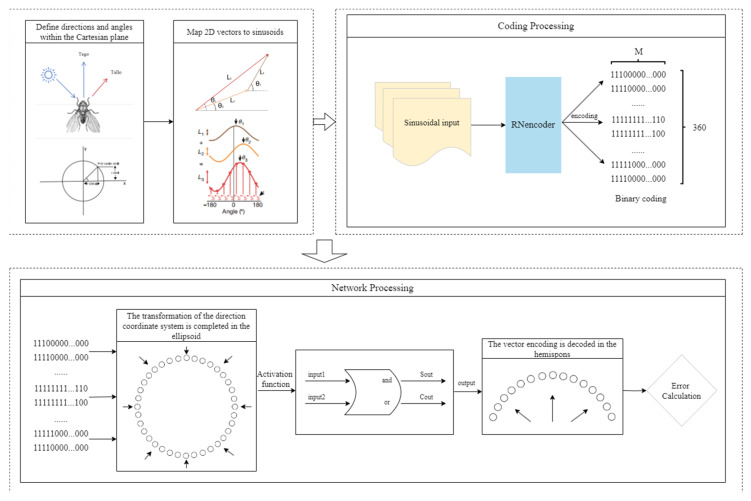
General idea.

At the core of our model lies a biomimetic EB-PB architecture. To facilitate the conversion of the directional space for varying particle sizes, we introduce a new model framework based on the half-adder principle. This model processes and learns the encoding of directional vectors, transforms the directional coordinate system within the ellipsoid structure, and decodes the transformed vector encoding in the protocerebral bridge. Finally, the proposed transformation method for ego–allo directional coordinates is assessed, with the overall concept illustrated in Figure 1.

We begin by clarifying the concept of the self–other coordinate system and providing its definition within a Cartesian framework. Next, we elaborate on the significance and methodology for transforming vectors in Cartesian space into sine curves. Building on the distinctive properties of sine curves, we then introduce our model’s encoding and decoding techniques. Finally, we test the proposed model and compare its performance with that of current state-of-the-art approaches.

### 3.2. Mathematical Modeling of the Ego–Allo Coordinate System

The distinction between egocentric and allocentric reference frames constitutes a cornerstone concept in the realm of spatial navigation, particularly in elucidating how animals monitor their movements through space. Understanding this distinction is crucial for developing robust models of spatial cognition and navigation. In the egocentric reference frame, the location of objects is described relative to the observer’s body or perspective. Conversely, in the allocentric reference frame, the location of objects is described relative to other objects or the environment [26].

The allocentric reference frame is established through the delineation of a stationary external axis, usually ascertained by visual landmarks or other environmental cues. The allocentric travel direction is subsequently defined as the angular relationship between the animal’s movement vector and this external reference axis. Mathematically, this relationship can be articulated as the summation of two angles: the angle between the head direction and the external reference axis, and the egocentric traveling angle in relation to the head direction. This mathematical framework facilitates the accurate tracking of movement direction in world-centered coordinates, irrespective of the animal’s orientation [38].

**Figure 2:**
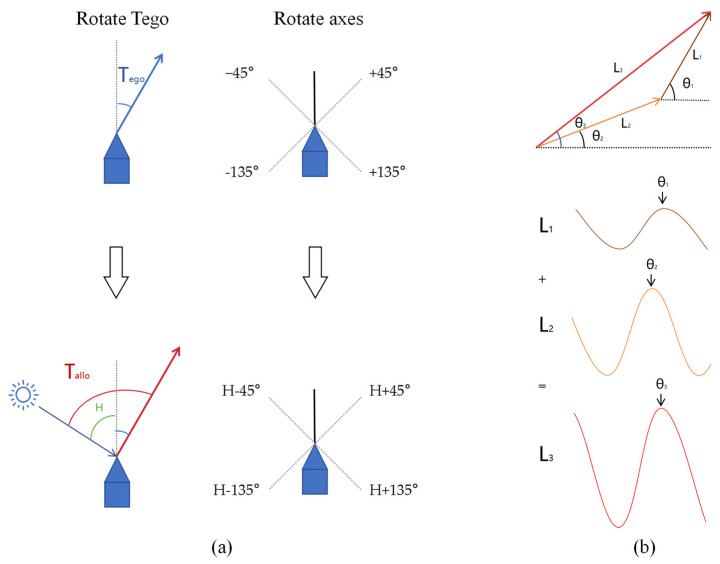
Coordinate model diagram illustrating the primary steps in transforming Drosophila’s egocentric travel direction into an allocentric framework. (**a**) The Drosophila’s movement direction vector is defined by its egocentric traveling angle $T_{ego}$, referenced to its head orientation. (**b**) A two-dimensional vector can be represented by a sinusoidal curve, adding a sinusoidal curve and then implementing vector addition.

Our mathematical framework addresses this limitation by explicitly defining two critical vectors in a two-dimensional coordinate system. The egocentric movement-direction vector represents movement relative to the animal’s head orientation rather than movement relative to an external reference axis., as shown in Figure 2a. This precise mathematical definition builds upon earlier work in path integration and vector navigation but provides a novel solution to the coordinate transformation problem.

To illustrate the transformation between egocentric and allocentric spatial representations, we constructed the coordinate model shown in Figure 2. In Panel (a), the diagram outlines how Drosophila’s head orientation (set as 0°) and external reference axes define the egocentric travel angle ($T_{ego}$) and the allocentric travel angle ($T_{allo}$). Specifically, the movement direction vector of Drosophila is defined by its egocentric traveling angle $T_{ego}$, which is referenced to its head orientation. This angle is projected onto four axes oriented at ±45° and ±135° relative to the head direction of the Drosophila. In this egocentric reference frame, the head orientation of the Drosophila represents 0°, and angles are considered positive in the clockwise direction. On the other hand, the allocentric traveling angle $T_{allo}$, referenced to an external coordinate system, can be derived by rotating the egocentric traveling direction $T_{ego}$ by adding H, the Drosophila’s allocentric heading angle, to reference the external world. Panel (b) presents the sinusoidal representation of a two-dimensional vector, where the phase indicates the direction, and the amplitude corresponds to the magnitude. This representation underpins the subsequent encoding process and neural translation mechanisms.

Following this approach, we further investigated the processing of directional vectors via bionic neural networks. We first present the definition of a vector in the Cartesian coordinate system:

Let the magnitudes of the two vectors be $r_{1}$ and $r_{2}$, with their corresponding angles being $\theta_{1}(\theta_{1}\in [0,2\pi ])$ and $\theta_{2}(\theta_{2}\in [0,2\pi ])$, respectively.

(1) $$\mathrm{For}\;\mathrm{vector}\;V_{1}:\left\{\begin{array}{l} x_{1}=r_{1}\cdot \cos(\theta_{1})\\ y_{1}=r_{1}\cdot \sin(\theta_{1}) \end{array}\right.,$$

(2) $$\mathrm{For}\;\mathrm{vector}\;V_{2}:\left\{\begin{array}{l} x_{2}=r_{2}\cdot \cos(\theta_{2})\\ y_{2}=r_{2}\cdot \sin(\theta_{2}) \end{array}\right.,$$

assume that the magnitude and angle of the resulting sum vector are $r_{sum}$ and $\theta_{sum}$, respectively. The calculation formulas are as follows:

(3) $$\begin{array}{l} r_{sum}=\sqrt{{\left(x_{1}+x_{2}\right)}^{2}+{\left(y_{1}+y_{2}\right)}^{2}}\\ \;\;\;\;\;\;\;=\sqrt{{\left(r_{1}\cos\theta_{1}+r_{2}\cos\theta_{2}\right)}^{2}+{\left(r_{1}\sin\theta_{1}+r_{2}\sin\theta_{2}\right)}^{2}}\\ \;\;\;\;\;\;\;=\sqrt{r_{1}^{2}+2r_{1}r_{2}\cos(\theta_{1}-\theta_{2})+r_{2}^{2}} \end{array},$$

(4) $$\begin{array}{l} \theta_{sum}=\arctan2(y,x)\\ \;\;\;\;\;\;\;\;=\arctan2(r_{1}\sin\theta_{1}+r_{2}\sin\theta_{2},r_{1}\cos\theta_{1}+r_{2}\cos\theta_{2}) \end{array},$$

where arctan2(y,x) calculates the azimuth angle from the origin to the point. Unlike the traditional arctangent function, arctan2(y,x) is capable of handling all four quadrants, ensuring that the return value lies within the range [−π,π]. For angle values less than 0, we add 2π to ensure that the overall range falls within [0,2π]. This adjustment standardizes the values to the desired interval.

In their study of directional navigation in Drosophila, Lyu et al. [26] revealed the biological foundation of vector computation mechanisms in the brain. They observed that neurons within the central complex of Drosophila perform vector calculations through sinusoidal activity patterns, mapping two-dimensional vectors onto periodic activity across distinct neuronal populations. Moreover, in signal processing and biomedical engineering, sinusoidal functions are widely utilized because of their distinctive periodic characteristics, making them particularly suitable for modeling rhythmic neural signals [39]. Brain signals, such as α, β, and θ waves in electroencephalograms (EEGs), often exhibit significant periodicity and oscillatory behavior. By employing a Fourier transform to decompose these signals into sinusoidal wave sequences, the frequency-band characteristics of brain activity can be extracted [40]. Thus, sinusoidal functions not only effectively capture the rhythmic nature of neural signals but also provide biological plausibility and interpretability in neural signal modeling [39,40]. This sinusoidal wave-based bioinspired model provides a fresh perspective for understanding the computational mechanisms of the brain. However, previous studies, such as Lyu et al. [26], have not integrated this bio-inspired framework with modern machine learning approaches, which limits its scalability and real-world applicability. Our work addresses this gap by proposing a hybrid architecture that combines sine encoding with machine learning, thereby achieving both biological plausibility and adaptive learning capabilities.

On the basis of this concept, we organically integrate directional vectors with sinusoidal functions and propose the use of sinusoidal functions to represent vector information. For a given vector, its encoding function is defined as:

(5) $$f(\phi )=r\cdot \sin(\phi -\theta +\frac{\pi }{2}),$$

where r represents the magnitude of the vector (for the purpose of result presentation, we define r∈[0,10]), θ is the angle of the vector (θ∈[0,2π]), and φ denotes the sampling point angle (ϕ∈[0,2π]). To fully represent the characteristics of the sinusoidal curve, we sample N points (taking N = 360) at equal intervals within the range [0,2π]. The angle of each sampling point is given by:

(6) $$\phi_{i}=\frac{2\pi i}{N},\;\;\;\;i=0,1,2,\ldots ,N-1,$$

Therefore, each sinusoidal signal can be represented by 360 equally spaced points, computed as follows:

(7) $$S=f(\phi_{i})=r\cdot \sin(\frac{2\pi i}{N}),\;\;\;\;i=0,1,2,\ldots ,N-1,$$

In summary, combining sinusoidal functions with bionic neural networks for vector encoding and summation offers a potentially more efficient, accurate, and biologically plausible solution for processing neural signals. This method leverages the periodic characteristics of sinusoidal curves to capture the dynamic patterns of neural activity, while employing neural networks to encode and compute complex vectors. This approach holds promise as an effective means for modeling complex brain activity patterns.

### 3.3. Direction Encoding Methods

After the directional vectors are converted into a sinusoidal pattern, they need to be represented in a format conducive to network processing. The accurate encoding of directional data is essential across various disciplines, including neuroscience research, autonomous robotics, and immersive virtual reality experiences [41]. Therefore, we propose a novel encoding method specifically designed to process directional information in neural models. This method addresses the core limitations of existing approaches, enhancing their effectiveness and adaptability in the representation of neural signals.

Our encoding methodology, the range-number encoder (RNencoder), employs a precise mathematical framework to convert continuous sinusoidal signals into discrete binary representations. This innovative approach addresses a core challenge in neural computation: representing continuous analog signals in a format that preserves essential information while remaining compatible with discrete neural processing systems.

The RNencoder uses a specialized mapping strategy to convert real numbers within the range of 0–N into binary sequences. The value of N is typically set equal to the magnitude of the vector r. For an input value x∈[0,N], the encoding function E(x) produces an M-dimensional binary vector, where M = kN, and k is the scaling factor:

(8) $$E(x)=[b_{1},b_{2},\ldots ,b_{M}],\;\;\;where\;b_{i}\in \left\{0,1\right\},$$

The binary elements are determined by:

(9) $$b_{i}=\left\{\begin{array}{c} 1,\;\;\;\;\;i\leq round(x)\\ 0,\;\;\;\;\;\;\;\;otherwise\;\;\; \end{array}\right\},$$

The encoding process can be visualized as a mapping from a continuous domain to a discrete binary space:

(10) $$[0,N]\to [0,M]\to {\left\{0,1\right\}}^{M},$$

The RNencoder (Equation (8)) converts continuous sinusoidal signals into sparse binary patterns, mimicking the sparse activity bumps observed in Drosophila EB [26]. Biologically, the 1-bit in the encoded vector corresponds to the active neuron clusters in EB-PB circuits, whereas the 0-bits reflect silent neurons that provide fault tolerance against noise.

Classical population coding techniques [42] have encountered difficulties in balancing resolution and computational efficiency, whereas our approach excels in achieving both through its binary representation framework. The encoder generates patterns in which the number of active bits directly aligns with the scaled input value, yielding a robust and easily comprehensible representation.

### 3.4. Bionic Model Similar to a Half-Adder Structure

We introduced a special model structure that processes directional information through a bionic design inspired by the anatomical organization of the Drosophila central complex. In the central complex of Drosophila, the egocentric heading direction is computed in the ellipsoid body and sent to the protocerebral bridge [11,24,34], whereas the body-centered translational direction is relayed to the nodulus [43]. Consequently, the raw data reaching the ellipsoid body represent the body-centered translational direction, whereas the travel direction expressed in the protocerebral bridge is egocentric. This section details our model architecture and its unique decoding mechanism.

**Figure 3:**
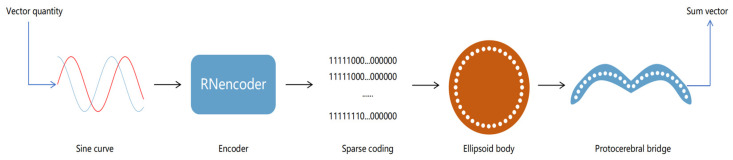
Network structure diagram depicting the structure of the input, hidden, and output layers along with the corresponding data flow.

The model architecture emulates the hierarchical structure of the Drosophila central complex. It comprises two layers, the ellipsoid body layer and the protocerebral bridge layer, each of which handles different aspects of directional information. The ellipsoid body layer functions as the computation layer, receiving encoded directional data and transforming the egocentric translational direction into allocentric heading direction via a simulated half-adder model. The protocerebral bridge layer acts as the decoding layer, receiving and decoding the heading information from the ellipsoid body layer into egocentric heading direction angles and velocities. The structural form is illustrated in Figure 3. The 360 real numbers sampled from two sinusoidal functions are encoded to form the input layer of the network, resulting in a total of 720 neurons. In the hidden layer, we process the input encoding via our proposed activation function. Finally, in the output layer, we obtain a summation sinusoidal function composed of 360 real-number encodings. By decoding these encodings, the resulting summation vector can be retrieved. The network architecture is detailed in Table 1.

**Figure 4:**
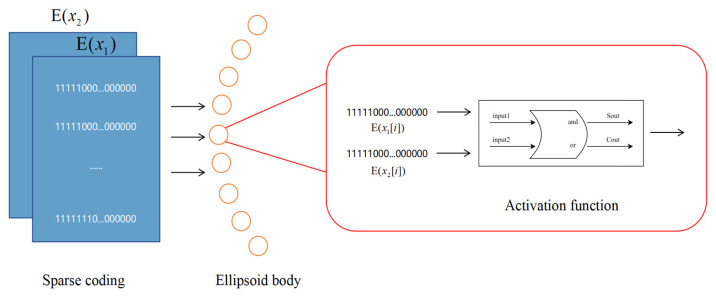
Hidden layer flowchart. The encoding processes for the two sinusoidal functions are computed within the hidden layer.

Figure 3 details the overall architecture of our proposed neural network. The input layer consists of 720 neurons generated by sampling two sinusoidal signals at 1° intervals over 360°. These inputs are converted into sparse binary patterns via the RNencoder. In the hidden layer (comprising 360 neurons), neurons are arranged in a circular pattern reminiscent of an ellipsoidal configuration and utilize a novel activation function based on half-adder logic. As depicted in Figure 4, each neuron simultaneously receives inputs from two neurons, processing the information via its unique activation function. This mechanism mimics the operation of a digital half-adder, integrating egocentric information and preparing it for conversion into allocentric coordinates. The output layer, consisting of 360 neurons, decodes the processed signals to reconstruct the allocentric motion direction vector.

The model’s bionic activation function is unique and processes paired inputs through a mechanism inspired by a digital half-adder. This novel approach represents a significant departure from traditional neural model design, which combines the precision of digital calculations with the inherent parallel processing capabilities of biological systems. As demonstrated by Sharp et al. [44], biological directional systems exhibit precise computational properties, and our activation functions are designed to simulate these properties.

The core innovation of our model lies in its biomimetic activation function, mathematically defined as follows: For input vectors $X_{1},X_{2}\in {\left\{0,1\right\}}^{M}$, the activation function f produces

(11) $$f(X_{1},\;X_{2})=[Y_{1},\;Y_{2},\;\ldots ,Y_{2M}],$$

where

(12) $$Y_{2i}=\left\{\begin{array}{c} 1,\;\;\;\;\;X_{1}[i]+X_{2}[i]>0\\ 0,\;\;\;\;\;\;\;\;\;\;\;\;\;otherwise\;\;\;\;\;\;\; \end{array}\right\},$$

(13) $$Y_{2i+1}=\left\{\begin{array}{c} 1,\;\;\;\;\;X_{1}[i]+X_{2}[i]>1\\ 0,\;\;\;\;\;\;\;\;\;\;\;\;\;otherwise\;\;\;\;\;\;\; \end{array}\right\},$$

**Figure 5:**
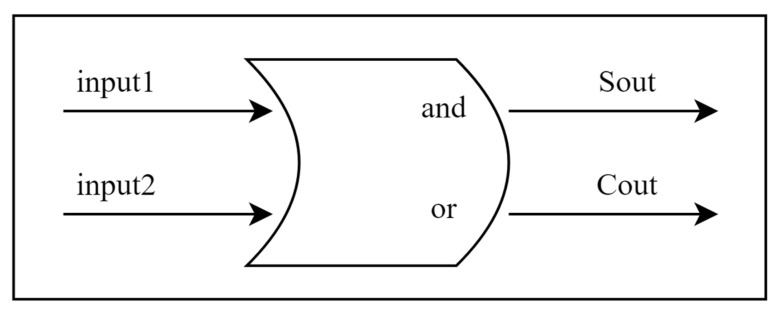
Half-adder-like model activation function. Each node has two inputs, “input1” and “input2”, which in turn produce two outputs, “Sout” and “Cout”, the core of which is modeled after the “and” and “or” logic of the half-adder.

This activation function is inspired by the principles of digital half-adders, representing a novel bridge between digital circuit design and biological neural processing. This approach is particularly significant because it combines the reliability of digital computation with the parallel processing capabilities of biological neural models [45]. Its structural diagram is shown in Figure 5.

Thus, Equation (11) can be further represented as:

(14) f(X1, X2)=[Y1, Y2, …,Y2M],   where Y2i=X1[i]|X2[i]Y2i+1=X1[i]^X2[i],

The AND/OR logic in the activation function is a simplified abstraction of the synaptic integration observed in Drosophila CX. Specifically:

The AND-like operation mimics phase-locked interactions between P-EN and E-PG neurons. Experimental studies indicate that P-EN neurons drive a systematic phase shift in the activity pattern of E-PG neurons through phase coupling [6]. This connection is analogous to the conditional dependency of an AND gate—downstream neurons are activated only when the input signals (such as speed and directional cues) are synchronized and meet a specific phase relationship. Notably, our design is inspired by this concept rather than representing a faithful reproduction of the model.

The OR-like operation reflects the convergence of multimodal sensory inputs (e.g., optic flow and polarized light) onto EB neurons, enabling robust directional encoding even if partial sensory modalities are disrupted [25].

**Figure 6:**
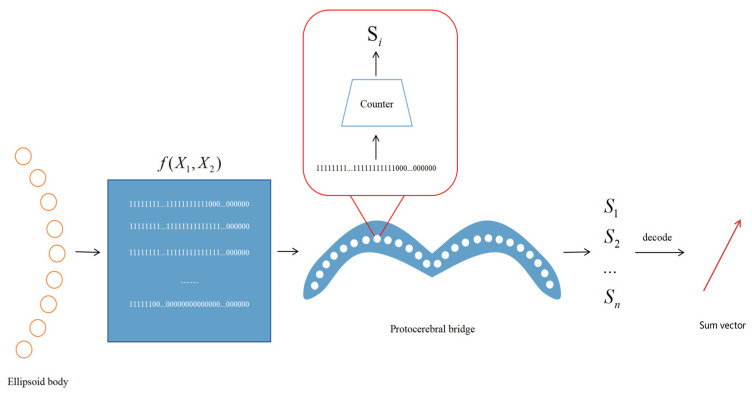
Decoding process of the network at the PB layer.

The neurons process the encoded data in the ellipsoid, transform the egocentric direction into the allocentric direction, and then transmit the data to the decoder for decoding to obtain the allocentric direction vector. The process is schematically illustrated in Figure 6.

Traditional methods often encounter noise and ambiguity, but our approach mitigates these issues by inversely converting encoded binary patterns into vector representations and then extracting directional information through phase—amplitude analysis.

In the decoding phase, the system processes the 360 real-number encodings from the output layer by statistically analyzing them to identify the encoding with the highest concentration of “1”s, which corresponds to the peak of the sinusoidal function within a single cycle. The relative position of this peak among the sampling points is determined and used to establish the maximum value point of the sinusoidal function. By combining this identified peak position with the sampling interval angle $\Delta \theta =\frac{2\pi }{N}$, the complete structure of the sinusoidal function is reconstructed.

The decoding process can be visualized as a mapping from a discrete binary space to a continuous domain:

(15) $$f(\theta )=r\cdot \sin(\theta +\theta_{offset}),$$

where $\theta_{offset}$ is the relative initial phase, which is calculated based on the basis of the positional offset of the maximum value point. r is determined by the difference between the maximum value and the minimum value of the summation sinusoidal function.

The system derives the phase and amplitude of the sine wave by analyzing the spatial characteristics of the sinusoidal pattern in continuous space. By analyzing these phase and amplitude values, the world-centered travel direction vector can be obtained.

Our pioneering neural architecture combines principles from digital circuit design with insights from biological neural systems to create a hybrid approach, offering new possibilities for directional information processing. In summary, the egocentric movement direction is projected onto four reference axes oriented at ±45° and ±135°. By appending the Drosophila’s concentric heading (H) to each of these axes and subsequently combining the corresponding vectors, an allocentric movement vector is obtained. Initially, we sample the vectors along the reference axes and encode them via sinusoidal functions. The resulting sine representations are then transformed into discrete binary codes, which are fed into the network. In the hidden layer, a specialized activation function processes these binary-coded inputs before passing them to the decoder, ultimately reconstructing the target allocentric vector.

### 3.5. Evaluation

To practically test the effectiveness of this method, we generated 200 pairs of two-dimensional directional vectors with magnitudes uniformly distributed between 0 and 10. A scaling factor, *k* = 20, was applied to obtain an encoding dimension *M* = 200. Each set of directional vectors was input into the system, and the model’s decoded results were compared with those obtained through algebraic calculations. The results demonstrated that even under challenging conditions, the system maintained accurate directional representation. This performance validates our design principles and reveals the broader application range of this method, from robotics to space navigation systems.

**Figure 7:**
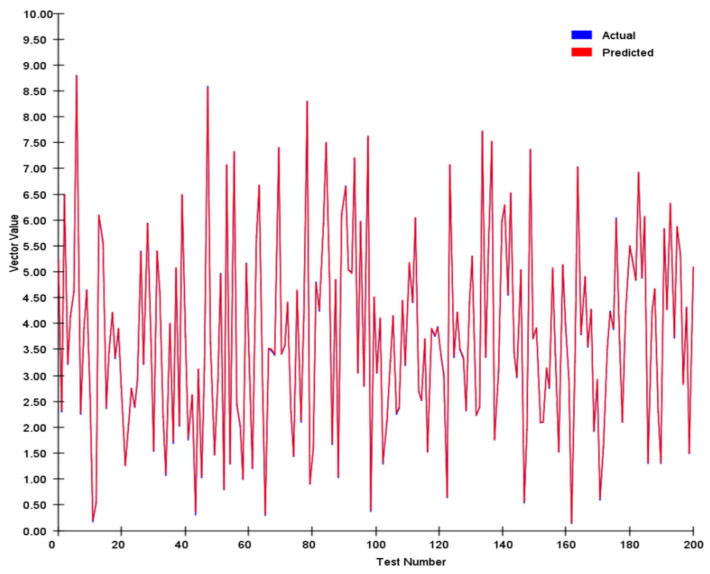
Vector magnitude analysis of two hundred pairs of random vectors.

**Figure 8:**
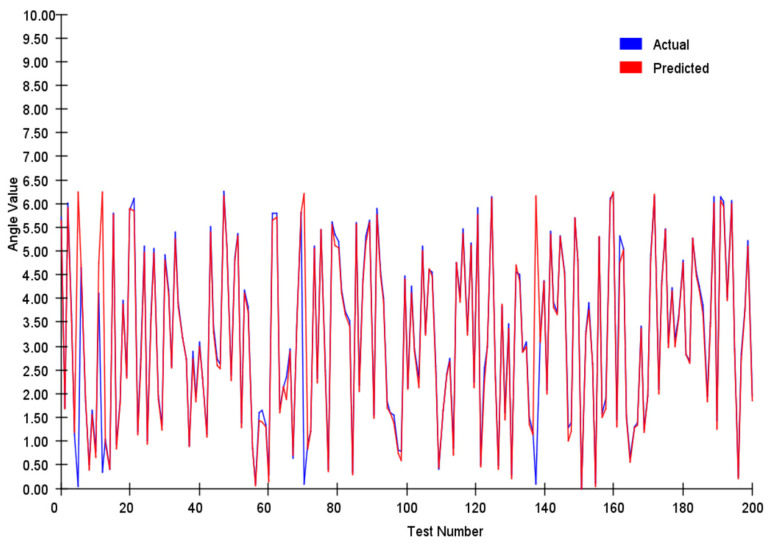
Vector angle analysis of two hundred random vectors.

We tracked and compared the vector’s module length and angle, and the results are shown in Figure 7 and Figure 8 below:

We conducted a comprehensive assessment of the direction vector encoding scheme through systematic testing. In tests involving 200 randomly generated vector pairs, the system demonstrated exceptional performance characteristics. Specifically, the average error in the vector magnitude was only 0.0342, whereas the angular achieved an average error of 0.1786. These metrics demonstrate that our encoding system achieved remarkable precision in preserving vector characteristic information.

In our angle comparisons, we observed a few instances of significant errors, primarily when the angles were near 0°. This issue is attributed mainly to the transition between 0° and 360° within the network. When actual values approach this boundary, any inherent network inaccuracies can amplify the prediction errors.

To further validate the effectiveness of the proposed approach, comparative experiments were conducted using a sparse autoencoder network, Long Short-Term Memory (LSTM), and Transformer models. The sparse autoencoder network is particularly adept at handling sparsely encoded information; as a variant of the standard autoencoder, it can more effectively learn sparse feature representations [46]. The network architecture is detailed in Table 2. Additionally, Transformer and LSTM models—both of which have received significant attention—were incorporated to assess the reliability of the quantitative experimental results.

We randomly generated 1000 samples as training data, where each sample includes input features (encoded values of magnitude and angle) and corresponding target outputs (magnitude and angle of the resultant vector). The input sample data were first passed through the input layer of the network. The data then went through two hidden layers, each utilizing the ReLU activation function. The first hidden layer consists of 1024 neurons, whereas the second hidden layer consists of 512 neurons. Finally, the output layer computed the results via a linear activation function.

**Figure 9:**
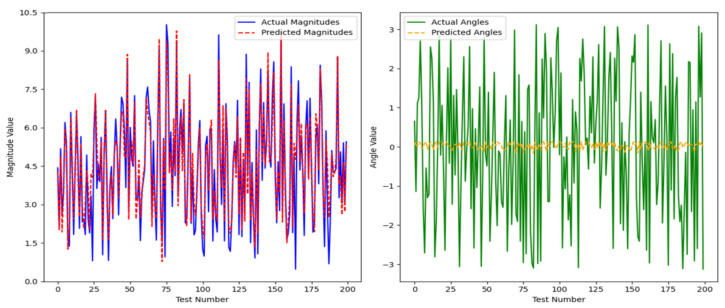
Vector error analysis of sparse autoencoder.

After model training, 200 randomly generated samples were used as test samples. The trained neural network was tested on these samples, and the results are presented in Figure 9.

The sparse autoencoder network was able to roughly learn the magnitude of the vector; however, the average error reached 0.0747. For the average angle, the error was as high as 3.194.

**Figure 10:**
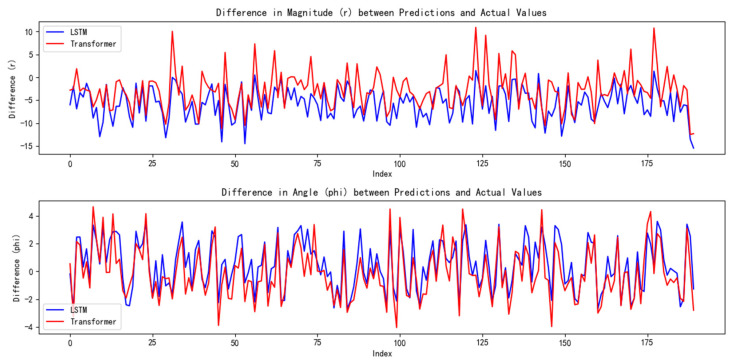
Error analysis plots for the LSTM and Transformer models.

Similarly, for the LSTM and Transformer models, an identical approach was adopted. A training set comprising 1000 randomly generated sample sets was created, and after the models were trained, an additional 200 random samples were generated for testing. The results are presented in Figure 10.

Through our calculations, the LSTM model yielded an average magnitude error of 5.8051 and an average angular error of 1.5759. In comparison, the Transformer model produced an average magnitude error of 4.1228 and an average angular error of 1.5253.

To better quantify the experimental results, we further introduced the root mean square error (RMSE) as a unified metric for measuring the magnitude of error. The RMSE is a widely used measure for assessing differences among numerical values, and it is calculated as follows:

(16) $$RMSE=\sqrt{\frac{\sum_{i=1}^{n}{\left(y_{pred}-y_{true}\right)}^{2}}{n}},$$

The experimental results are summarized in Table 3 below.

The experimental results indicate that our model demonstrates superior accuracy compared with both the sparse autoencoder—known for its effective handling of sparse coding—and the currently popular Transformer and LSTM models.

**Figure 11:**
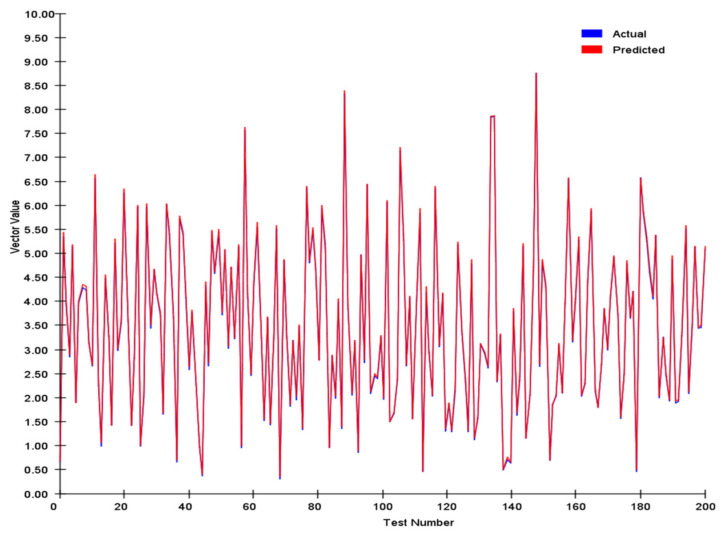
Analysis of the error magnitudes for noisy vectors.

**Figure 12:**
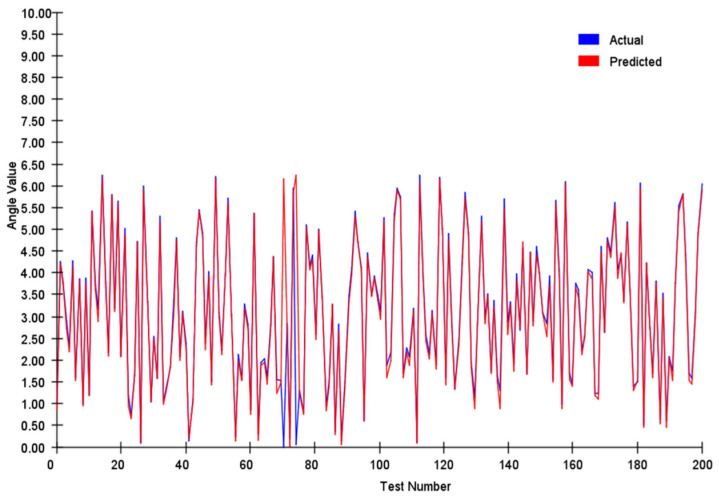
Analysis of the error angles for noisy vectors.

To validate the model’s robustness, we augmented the original experiments by incorporating noise simulation. The noise of varying magnitudes was added to the input vectors, and the network’s performance was evaluated under these conditions. The results of these tests are presented in Figure 11 and Figure 12.

In the noise test, the average modulus length error of the model was 0.0813 and the angular error was 0.1563. These results are lower than those achieved by other models, highlighting the robustness and superior performance of our approach under noisy conditions.

**Figure 13:**
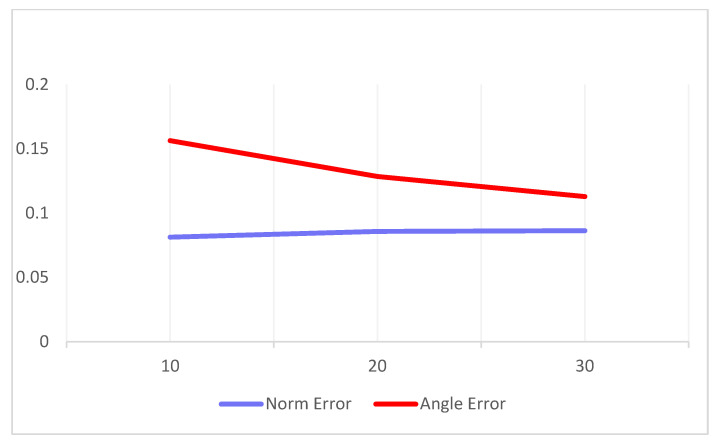
Error variation analysis across different input scales.

In addition, we conducted further experiments using more complex and longer input vectors. Specifically, we increased the number of magnitude inputs to 20 and 30, to assess the model scalability. The variation in the average errors corresponding to these increased input lengths is illustrated in Figure 13. The results indicate that although the computational cost increased linearly with the number of input samples, the output accuracy remained stable, thereby demonstrating the scalability of our approach.

These experimental findings carry significant theoretical and practical implications. In biological navigation systems, transitioning between egocentric and allocentric reference frames is a fundamental challenge. Our experimental approach achieved a high-precision conversion between these two spatial systems, thereby not only validating potential computational mechanisms observed in nature but also offering a novel solution for spatial cognition in artificial intelligence.

### 3.6. Computational Complexity Analysis

To evaluate the practical applicability of our model, we analyzed its computational complexity in terms of time and space requirements.

(1) Time Complexity.

The model’s forward propagation involves three layers (Table 1), with time complexity dominated by matrix operations:

(17) $$O(n_{in}\cdot n_{hidden}+n_{hidden}\cdot n_{out}),$$

where $n_{in}=720$, $n_{hidden}=360$, and $n_{\mathrm{out}}=360$. For each input sample, the total number of operations is 720 × 360 + 360 × 360 = 388,800 operations.

(2) Space Complexity.

The model requires storing:

Parameters: 388,800 weights (1.6 MB in float32).

Activations: 360 + 360 = 720 neurons per sample.

(3) Scalability.

For input neurons d⋅M, the model scales linearly:

Time: $O(d\cdot M\cdot n_{hidden}+n_{hidden}\cdot n_{out})$.

Space: $O(d\cdot M\cdot n_{hidden}+n_{hidden}\cdot n_{out})$.

Our model achieves linear time and space complexity through a shallow, sparsely encoded architecture, making it highly suitable for real-time applications in resource-constrained systems.

## 4. Discussion

Existing CX-based models face a fundamental trade-off: biological fidelity (e.g., Varga and Ritzmann’s SNN [47]) sacrifices computational efficiency, whereas anatomical precision (e.g., Turner-Evans’ phase coupling [34]) limits adaptability. Our model transcends this trade-off through a hybrid design—integrating digital half-adder logic with Drosophila CX’s functional principles. First, we propose a hybrid digital–biological computational framework that integrates the deterministic half-adder logic from digital circuits with the Drosophila CX circuitry, particularly the EB-PB architecture. This integration enables precise computation while maintaining biological plausibility, effectively addressing the longstanding trade-off between accuracy and biological fidelity in existing models [6,26].

Second, we introduce a novel sparse binary encoding mechanism called the RNencoder. Unlike the continuous phase encoding utilized in prior studies [26], our approach converts sinusoidal signals into sparse binary patterns, mimicking the sparse activity bumps observed in the Drosophila EB. Compared with traditional methods, this design significantly reduces noise sensitivity.

Finally, inspired by the Drosophila central complex circuits, we abstract key computational principles from the Drosophila CX while simplifying certain biological details to design this network. This facilitates a bioinspired transformation between egocentric and allocentric coordinate systems (ego–allo). These contributions collectively represent a significant advancement in computational efficiency, accuracy, and robustness within the field.

The significance of this research extends beyond mere technical achievements. The successful demonstration of effective spatial reference frame transformation addresses a fundamental problem in neuroscience and cognitive science: how biological systems seamlessly integrate and transform different spatial representations. The performance of our model suggests that relatively simple and elegant computational mechanisms may underlie these complex cognitive processes. This insight may help bridge the gap between neurobiological observations and theories of spatial cognitive computing [33].

These findings also have broad implications for the development of artificial systems that must operate in complex space environments. The high accuracy and reliability of our coding scheme suggest that it can be used as a basis for more complex spatial reasoning systems. In robotics, autonomous navigation, or virtual reality applications, the ability to effectively transition between different spatial reference frames while maintaining high accuracy is critical to system performance and reliability.

However, several limitations and areas for future improvement should be noted:

While the half-adder-like activation function captures essential computational principles of the Drosophila CX, it simplifies biological complexity. For example, synaptic plasticity and neuromodulation are not explicitly modeled. Future work could integrate dynamic synaptic adaptation (e.g., STDP) to better align with biological observations.

As demonstrated by Burak and Fiete [48], neural noise can significantly impact spatial encoding accuracy. The error in handling the 0°/360° boundary underscores a critical divergence from biological systems. In the Drosophila CX, circular attractor networks seamlessly manage periodic continuity [11], whereas our static model experiences discrete jumps at the boundary. Future work could incorporate ring connectivity and dynamic attractor mechanisms to bridge this gap, thereby enhancing both accuracy and biological plausibility.

Our testing was limited to two-dimensional vectors, whereas real-world navigation often involves three-dimensional spatial transformations. Future work should extend this model to handle three-dimensional spatial representations, as suggested by recent studies on three-dimensional spatial navigation in flying animals.

The computational cost increases significantly with increasing encoding resolution, which might pose challenges for real-time applications. This limitation echoes similar challenges faced in implementing bioinspired navigation systems.

Looking forward, several promising directions for future research have emerged:

1. Compared with that of Drosophila CX, the higher neuron count reflects a trade-off between biological fidelity and computational feasibility. Biological systems achieve efficiency through sparse activity and adaptive plasticity, whereas our static network compensates with redundancy. Integrating bioinspired sparsity and plasticity could reduce dimensionality while preserving performance.
2. Extending the system to simultaneously accommodate multiple reference frames would more faithfully capture the flexibility inherent in biological navigation systems.
3. Developing more robust error-correction mechanisms, inspired by the redundancy principles observed in biology, could further enhance system resilience.

## 5. Conclusions

In this study, we presented a comprehensive framework for transforming egocentric spatial representations into allocentric coordinates. Our proposed method integrates sinusoidal signal encoding, sparse binary pattern generation, and a half-adder logic-based activation function, effectively bridging the gap between egocentric and allocentric perspectives. Extensive experimental evaluations demonstrate the superior performance of our approach. Under ideal conditions, our model achieved a magnitude RMSE of 0.0400 and an angular RMSE of 0.6191, outperforming other models such as sparse autoencoder, LSTM, and Transformer networks even under noisy inputs and varying input scales. These quantitative results validate the robustness and accuracy of our design. The innovative aspects of our research not only deepen our understanding of spatial encoding in biologically inspired systems—particularly reflecting mechanisms observed in the Drosophila central complex—but also underscore the feasibility of applying such designs in practical navigation and neural computation tasks. While the current model shows strong performance, we acknowledge limitations in handling boundary discontinuities and its current restriction to two-dimensional vector transformations. Future work will focus on integrating adaptive correction mechanisms for angular discontinuities and extending the framework to three-dimensional spatial representations. Overall, our findings emphasize the significant potential of biologically inspired models in advancing both artificial intelligence and computational neuroscience, paving the way for more sophisticated spatial reasoning systems in robotics and autonomous navigation.

## Figures and Tables

**Table 1:** The structure of the neural network.

Structure	Neuron Counts
Input Layer	720
Hidden Layer	360
Output Layer	360

**Table 2:** The structure of the sparse autoencoder network.

Structure	Neuron Counts	Activation Function
Hidden Layer 1	1024	ReLU
Hidden Layer 2	512	ReLU

**Table 3:** Experimental results of different models under the same experimental conditions.

Structure	Our Model	Sparse Autoencoder	LSTM	Transformer
Magnitude RMSE	0.0400	0.0936	6.6936	4.5333
Angle RMSE	0.6191	4.0014	5.7189	3.8934

## Data Availability

According to the confidentiality of the funding project, the codes and data supporting the survey results of this paper are not disclosed at present because the research has not been completed. You can request a copy from the author at zzuwhd@gs.zzu.edu.cn.

## Abbreviations

The following abbreviations are used in this manuscript:

CX	central complex
PB	protocerebral bridge
FB	fan-shaped body
EB	ellipsoid body
EB-PB	ellipsoid body–protocerebral bridge
ego–allo	egocentric-to-allocentric
PCA	Principal Component Analysis
CCA	Canonical Correlation Analysis
TLS	total least squares
WTLS	weighted total least squares
E-PG	ellipsoid body–protocerebral bridge–gall
P-EN	protocerebral bridge–ellipsoid body–nodulus
EEG	electroencephalogram
RNencoder	range-number encoder
2D	two-dimensional
